# Tet3-mediated DNA demethylation is essential for maintaining the dedifferentiation capacity of mammalian Müller glia

**DOI:** 10.3389/fnmol.2025.1628860

**Published:** 2025-07-17

**Authors:** Erick J. Martinez-Colin, Ivonne Lezama, Lenin D. Ochoa-de la Paz, Monica Lamas

**Affiliations:** ^1^Departamento de Farmacobiología, Mexico City, Mexico; ^2^Centro de Investigación sobre el envejecimiento, Mexico City, Mexico; ^3^Laboratorio de Neurobiología Molecular y Celular de la Glía, Facultad de Medicina, Departamento de Bioquímica, UNAM, Mexico City, Mexico; ^4^Unidad de Investigación APEC-UNAM, Asociación Para Evitar la Ceguera en México I.A.P., Mexico City, Mexico

**Keywords:** retina, Müller glia, epigenetics, dedifferentiation, neuronal differentiation

## Abstract

Müller glia (MG) are retinal resident cells with diverse functions, including reprograming and regeneration in certain species. While the mammalian retina possesses molecular mechanisms for MG dedifferentiation and neuronal differentiation, it fails to generate neural progenitors *in vivo*. We previously proposed that an epigenetic barrier, driven by DNA methylation, may prevent complete MG reprograming in response to damage. DNA demethylases, such as Ten-Eleven Translocation (TET) and Growth Arrest and DNA Damage-Inducible Protein 45 (GADD45) families, are induced by damage and play roles in cellular reprograming and regeneration. Tet enzymes are regulated by metabolic factors (iron, vitamins A and C) and respond to environmental stimuli. Here, we investigated DNA demethylation as a regulatory signal for MG dedifferentiation and neuronal differentiation in response to N-methyl-D-aspartate (NMDA), a neurotransmitter used to model retinal neurodegeneration. Using mouse primary cultures and antibodies against methylated (5mC, MeCP2) and unmethylated (5 hmC, H3K4Me3) DNA, we analyzed MG epigenetic changes under control, vitamin-supplemented, and NMDA-stimulated conditions. We also assessed DNA demethylase expression and key reprograming genes (*Ascl1, Lin28, Nestin*). Vitamin A and C increased 5hmC levels but did not upregulate Tet enzymes or reprograming genes. In contrast, NMDA increased *Tet3* and reprograming gene expression. *Tet3* knockdown led to a rapid 5mC increase and impaired NMDA-induced upregulation of *Ascl1, Lin28*, and *Nestin*, suggesting its critical role in MG dedifferentiation. Conversely, Tet*3* overexpression induced morphological changes and early neuronal marker expression. These findings identify Tet3 as a key epigenetic regulator of MG reprograming and a potential target for retinal regeneration strategies.

## Introduction

Müller glia (MG) of retinal-regenerative species such as zebrafish, and to a certain extent mammalian Müller cells, exhibit a dedifferentiation capacity that that enables them to acquire a “progenitor-like” phenotype and further differentiation into a functional neuronal phenotype in response to damage (reviewed in Hamon et al., 2016). High-throughput profiling methods, such as single-cell transcriptomic analysis, have enabled the identification of a core transcriptome that potentially underlies Müller cell function in both mice and humans (Roesch et al., 2008; Lukowski et al., 2019; Voigt et al., 2021). Subsequent studies have reinforced the importance of epigenetic marks, such as DNA methylation or histone posttranslational modifications, in retinal development and physiology (Corso-Díaz et al., 2018). Specifically in Müller cells the epigenomic plasticity in relation to DNA methylation status has been shown to underly their neuronal regeneration capacity (Lahne et al., 2020; Dvoriantchikova et al., 2019a) and their response to injury and aging (Lin et al., 2019). In both zebrafish and mammals, DNA demethylation processes mark the initial steps of MG reprograming toward retinal regeneration (Powell et al., 2013; Dvoriantchikova et al., 2019a). DNA demethylation can occur either passively due to a deficiency in DNA methyltransferase enzymatic activity or, in an active manner, driven by DNA demethylase activities. Proteins involved in DNA demethylation include, among others, the non-enzymatic Growth arrest and DNA-damage-inducible protein 45alpha and 45beta (Gadd45a and Gadd45b), as well as the Ten-Eleven Translocation (Tet1, Tet2 and Tet3) protein family, which convert 5-methylcytosine (5 mC) to 5-hydroxymethylcytosine (5-hmC) (Carey et al., 2011). Specifically, Tet proteins are hydroxylases dependent on α-ketoglutarate, Fe (II) and molecular oxygen and their activity can be regulated by metabolic effectors like vitamins A and C (Kohli and Zhang, 2013; Hore et al., 2016).

In this study, we employed specific antibodies targeting epigenetic marks associated with a transcriptional-permissive, unmethylated DNA (5-hydroxymethylcytosine, H3K4me3) or a transcriptional-restrictive, methylated DNA (5-methylcytosine, MeCP2) states, to evaluate epigenetic landscape alterations in murine MG cells in response to a well-documented MG dedifferentiation signal, N-methyl-D-aspartate (NMDA) (Karl et al., 2008; Ramírez and Lamas, 2009; Takeda et al., 2008; Reyes-Aguirre et al., 2013; Xiao et al., 2017; Carapia et al., 2023). Our findings provide evidence that, upon exposure to 100 μM NMDA, 100 nm vitamin A or 100 μM vitamin C, the epigenetic landscape of murine MG cells undergoes a transition from a restrictive to a permissive state associated to a global increase on DNA demethylation. However, only upon NMDA exposure, the epigenetic landscape alteration could be associated to the induction of the expression of Tet3 and pluripotency genes *Lin28*, *Ascl-1* and *Nestin*. siRNA-mediated knockdown approaches demonstrate that Tet3 expression is essential for maintaining the dedifferentiation capacity of mammalian Müller glia.

Cellular dedifferentiation and subsequent neuronal differentiation are closely linked processes that, in the retinas of regenerative species, can lead to full functional regeneration (Thummel et al., 2008). The reasons underlying the very low efficiency of neuronal differentiation of mammalian Müller glia remain unknown but have been associated to a repressive chromatin state of genes required for the development of early-born retinal neurons (Dvoriantchikova et al., 2019b). Thus, it has been proposed that DNA demethylase activities, together with other concurrent processes, could restore the adult mammalian MG neuronal differentiation competence (Dvoriantchikova et al., 2019a). We here, using a plasmid-mediated overexpression approach, were able to link Tet3 upregulation with morphological alterations and an increase in early neuronal markers expression in cultured mammalian MG. Overall, these findings identify Tet3-mediated DNA demethylation as a key epigenetic regulator of mammalian MG reprograming and a potential target for retinal regeneration strategies.

## Materials and methods

### Animal subjects

Postnatal (8–12 days) C57BL/6J mice (RRID:IMSR_ JAX:000664) were used for all experiments. The laboratory animals were treated and handled in strict accordance with the Association for Research in Vision and Ophthalmology (ARVO) Statement for the Use of Animals in Ophthalmic and Vision Research, and the guidelines of the internal animal care committee (CICUAL-CINVESTAV; Project number: 0259-17).

### Cell culture

Müller glia cell cultures were obtained from mice ranging from 8 to 12 post-natal days as previously described (Carapia et al., 2023). Briefly, mice were beheaded and the eyes enucleated. Eyes were placed in DMEM (Gibco) media in the dark for an overnight incubation with agitation at room temperature. The next day an enzymatic digestion was performed, using DMEM containing 0.1% trypsin and 70 IU/mL collagenase (Sigma Chemical Co., St. Louis, MO) for 30 min at 37°C. The digestion was stopped with DMEM supplemented with 10% FBS (Gibco). The eyes were transferred to a Petri dish containing DMEM supplemented with 10% FBS (DMEM-FBS 10%). The connective tissue was removed, and the eyes were cut above the *ora serrata* to obtain retinal cups. Retinal tissue was removed from the cups, discarding vitreous and RPE in the process. Retinas were dissociated by pipetting in an Eppendorf tube filled with 2 mL DMEM-FBS 10%. Then, dissociated cells from 8 to 10 retinas were seeded into a six-well plate with DMEM-FBS 10% and penicillin-streptomycin 1% and placed in an incubator at 37°C and 5% CO_2_. The cells were allowed to attach for 24 h then washed with PBS 1X to eliminate non-adherent cells and debris, and the media was replaced. The cells were kept in culture and the medium was replaced after 4–5 days, after this, the medium was changed every 3 days. The cells were maintained for 2 weeks or until they were confluent.

### Pharmacological treatments

Müller glia was seeded at 1.5 × 10^5^ cells per well in a six well plate for qPCR experiments and at 3 × 10^4^ for each coverslip for immunofluorescence experiments. NMDA stimulation consisted in 100 μm of NMDA (Sigma Chemical Co., St. Louis, MO) for 6 h followed by cell culture media replacement, as previously described (Carapia et al., 2023). For vitamin treatment we used 100 nm of retinoic acid (vitamin A) (Sigma Chemical Co., St. Louis, MO) (Hore et al., 2016) or 100 μm of ascorbic acid (vitamin C) (Sigma Chemical Co., St. Louis, MO) (Blaschke et al., 2013) and the combination of both treatments for 6 h all of them, followed by culture media replacement.

### qPCR

RNA was extracted with Trizol (Sigma Chemical Co., St. Louis, MO), next cDNA was synthesized with RevertAid First Strand cDNA Synthesis Kit (Thermo Fisher Scientific) using up to 1,000 ng of RNA to normalize between samples. qPCR was performed on a Rotor Gene Q (Invitrogen) using a SYBR-green based assay to evaluate the expression of *Tet1, Tet2, Tet3, Gadd45a, Ggadd45b, Pax6, Ascl1, Lin28, Cralbp, Tuj1, and Nestin* (Table 1). We used a PCR protocol consisting of the conditions indicated on Table 2. Normalization was performed relative to *Gapdh* using delta delta Ct relative expression method (Livak and Schmittgen, 2001).

**TABLE 1:** qPCR primers.

Gene	Sequence
*Ascl1*	F: GCAACCGGGTCAAGTTGGT R: GTCGTTGGAGTAGTTGGGGG
*Cralbp*	F: ACTGGCACTGTGAAGAAGTGACCT R: AGTCAGCAGGCAGGATGTTCTCAT
*GAPDH*	F: ACTGGCATGGCCTTCCGTGTTCCTA R: TCAGTGTAGCCCAAGATGCCCTTC
*Gadd45a*	F: CCTGCACTGTGTGCTGGTGA R: CCACTGATCCATGTAGCGACTTTC
*Gadd45b*	F: CCTGGCCATAGACGAAGAAG R: AGCCTCTGCATGCCTGATAC
*Nestin*	F: AGGAGAAGCAGGGTCTACAGAG R: AGTTCTCAGCCTCCAGCAGAGT
*Pax6*	F: TTTAACCAAGGGCGGTGAGCAG R: TCTCGGATTTCCCAAGCAAAGATG
*Tet1*	F: AGCCTGTTCCTCGATGTGG R: CAAACCCACCTGAGGCTGTT
*Tet2*	F: GCCATTCTCAGGAGTCACTGC R: ACTTCTCGATTGTCTTCTCTATTGAGG
*Tet3*	F: GGTCACAGCCTGCATGGACT R: AGCGATTGTCTTCCTTGGTCAG
*Tuj1*	F: CTGGAGCGCATCAGCGTATAC R: ATCTGGTGCGTGAGCTCAGG

**TABLE 2:** qPCR cycling conditions.

Cycle step	Temperature	Time	
Initial denaturing and heating	95°C.	15 min	
Denaturation	95°C.	15 s	Cycle
Annealing	60°C.	30 s	
Extension	72°C.	30 s	
Melting curve with default settings configuration			

### Tet 3 siRNA knock down

Tet3 knockdown was performed using two different small interfering RNAs (siRNAs): a Tet3-specific siRNA (Santa Cruz Biotechnology) and a non-targeting Control siRNA A (Santa Cruz Biotechnology). Transfection was carried out using the reverse transfection method with siPORT NeoFX transfection reagent (Invitrogen).

Briefly, siPORT NeoFX was mixed with Opti-MEM (Gibco) and incubated for 10 min at room temperature to allow lipid complex formation. siRNA was resuspended in Opti-MEM and added to the siPORT solution, followed by an additional 10-min incubation to facilitate siRNA-lipid complex formation. The siRNA-siPORT mixture was then distributed into each well of a six-well plate. Müller glia cells (1.5 × 10^5^ per well) were seeded for qPCR analysis, while 3 × 10^4^ cells per well were plated onto coverslips for immunofluorescence experiments. Cells were harvested for downstream analyses 24 h post-transfection.

### Tet 3 overexpression

Müller cells were seeded onto 6-well plates at a density of 2.5 × 10^5^ cells per well and incubated for 24 h. Two hours prior to transfection, the culture medium was changed to Optimem. For Tet3 overexpression, we used a previously reported Tet3 plasmid by Dr. Yi Zhang, pcDNA-Flag-Tet3 (Addgene plasmid #60940;^1^ RRID:Addgene_60940) (Wang and Zhang, 2014); a pBluescript II KS(+) plasmid was used as a control plasmid for the transfection procedure. Lipofectamine 3,000 reagent (Thermo Fisher Scientific) was used for transfection, with 5 μL of Lipofectamine per well, following the kit’s instructions. Six hours post-transfection, the lipid complex medium was replaced with DMEM supplemented with 10% fetal bovine serum (FBS) without antibiotic-antimycotic to avoid cytotoxicity. After 24 h, the medium was replaced with Neurobasal supplemented with B27 and N2 for neural culture, and 10% antibiotic-antimycotic. Tet3 overexpression was assessed 24 h post-transfection, and the cells were processed for qPCR and immunofluorescence 7 days post-transfection.

### Immunofluorescence for nuclear and cytoplasmic markers

Cells cultured on coverslips were fixed with 4% paraformaldehyde (PFA) for 15 min. The coverslips were then washed with PBS for 10 min and incubated with Tris-glycine (10 mM/100 mM) for 10 min to quench the PFA fixation. Permeabilization was carried out with 0.1% Triton X-100 in PBS for 10 min, followed by three PBS washes, 10 min each with agitation. For 5mC and 5hmC staining, a nuclear antigen retrieval process was necessary. The coverslips were incubated with 4N HCl at 37°C for 10 min, after which the HCl was removed, and Tris-HCl (pH 9.0) was added to neutralize the acidity for another 10 min at 37°C. Three PBS washes, 10 min each with agitation, were performed to remove any remaining acid residue. Subsequently, all coverslips, regardless of treatment, were blocked with normal goat serum (5%), Triton X-100 (0.01%) in PBS for 30 min, followed by three PBS washes, 10 min each with agitation. Primary antibody solutions listed in Table 3 were prepared in normal goat serum (5%), Triton X-100 (0.01%) in PBS. The primary antibody dilutions were: 5mC (1:200), 5hmC (1:200), MeCP2 (1:250), H3K4Me3 (1:200), Lin28 (1:300), Glutamine synthetase (1:300), Vimentin (1:200), FLAG (1:200), Nestin (1:250), and Beta III Tubulin (1:300). Primary antibodies were applied to cover the entire surface of the coverslips and incubated overnight (18 h) in the dark at 4°C.

**TABLE 3:** Antibodies used for this study.

Antibody	Brand	# Catalog
5mC	Abcam	Ab10805
5hmC	Abcam	Ab214728
H3K4Me3	Merck	07-473
MeCp2	Sigma	M6818
Glutamine Synthetase	Abcam	Ab73593
Lin28	Abcam	Ab46020
Nestin	Abcam	Ab6142
Tet 3	Genetex	GTX121453
Beta III tubulin	Santa cruz	SC80005
FLAG peptide	Merck	F7425
Alexa 488 donkey α rabbit	Invitrogen	A21206
Alexa 488 goat α mouse	Invitrogen	A11001
Alexa 568 donkey α rabbit	Invitrogen	A10042
Alexa 568 goat α mouse	Invitrogen	A11031
Cy5 goat α mouse	Abcam	Ab6563
Cy5 goat α rabbit	Abcam	Ab6564

The following day, coverslips were washed with PBS (1X) three times, 10 min each with agitation. Secondary antibody solutions were prepared with normal goat serum (5%) and Triton X-100 (0.01%) in PBS, using Alexa Fluor 488 and Alexa Fluor 568 at a 1:1,000 dilution, and Cy5 at a 1:800 dilution. Secondary antibodies were incubated with the coverslips for 1 h at room temperature in the dark. Afterward, the coverslips were washed with PBS (1X) three times, 10 min each with agitation. For counterstaining, DAPI was used at a 1:1,000 dilution and incubated for 2 min. Rhodamine-phalloidin was used at a 1:1,000 dilution in PBS (1X) with 1% BSA and incubated for 5 min. Finally, coverslips were mounted with ProLong Diamond Antifade (Thermo Fisher Scientific).

### Confocal imaging

Cell imaging was obtained using a ZEISS Axiovert 4°C/40 CLF inverted Fluorescent Microscope (Carl Zeiss, AXIO VISION Rel. 4.8 software) and an LSM 800 confocal system coupled to an inverted Axiovent AX10 microscope (Carl Zeiss, ZEN blue edition). Confocal images were constructed by Z-stack to analyze individually, and a projection based upon Z-stacks for images showed in this paper. Quantification of images and fluorescence levels was obtained using 3 biological replicas with at least 5 different fields and a minimum of 30 cells counted per replica. For nuclear imaging we used original data colorless images, identified nucleus as primary object using 5mC or DAPI as the reference for nucleus region, measured the reference object fluorescence. Then, we created a mask using reference staining to measure the same defined areas for each of the nuclear marks, then measured both fluorescence of the reference signal and for the masked images for 5hmC, MeCP2 and H3K4Me3. Background signal was subtracted using fluorescence thresholding, the fluorescence values were then normalized in-software relative to the area measured to obtain fluorescence values that included all the nuclear area, considering both the nuclear foci and the surrounding chromatin. Individual foci or their specific intensity was not evaluated.

For cell body fluorescence, actin staining was used to define the cell area, then masked the images for the defined areas. Fluorescence of Cralbp, Beta III tubulin, Glutamine synthase, Lin28, and FLAG was then measured and normalized to cell area, background signal was subtracted using fluorescence thresholding. Afterward, all fluorescence values were transformed into normalized data via the minimal-maximal method to obtain values between zero and one.

### Statistical analysis

Unless stated otherwise, experiments were performed as three biological replicates with two technical replicates each. Experiments comparing NMDA with control conditions were tested with a T student for independent samples. Experiments with three or more groups were tested with a one-way ANOVA and a Dunnett multiple comparison test to determine the treatment different from control. Graphpad Prism 8.0.1 was used to generate the graphs and the statistical analysis.

## Results

### NMDA induces alterations in the epigenetic landscape of cultured MG

**FIGURE 1:**
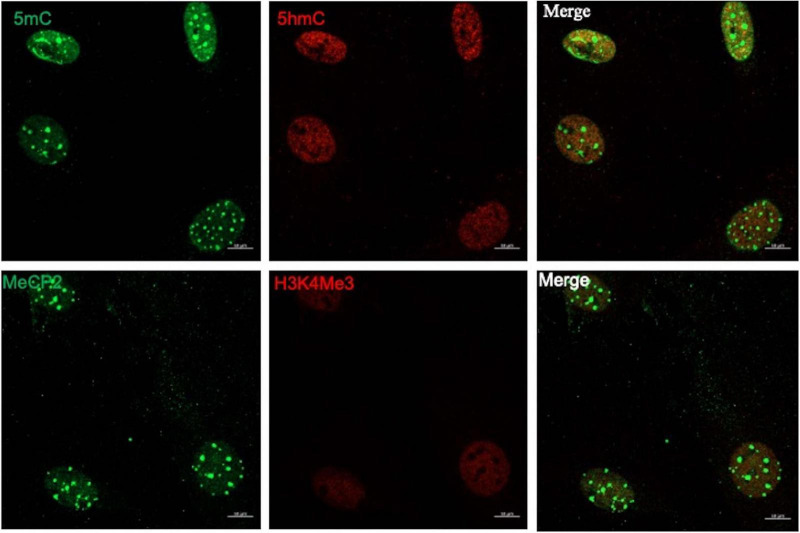
The MG epigenetic landscape. Representative images of confocal imaging of immunofluorescence to assess the chromatin epigenetic landscape in cultured MG, using both repressive (5mC and MeCP2; green) and active (5hmC and H3K4Me3, red) chromatin markers. Calibration bar: 10 μm.

**FIGURE 2:**
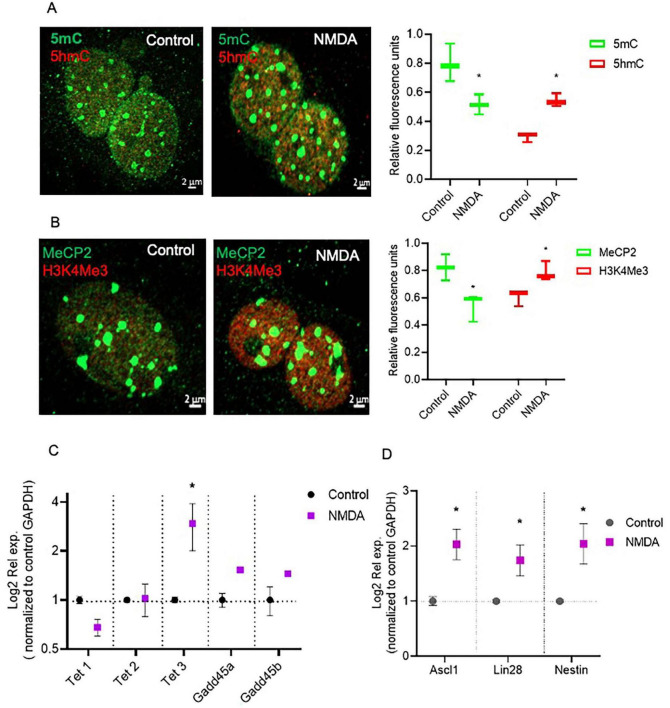
NMDA induced DNA demethylation changes that correspond with transcriptional changes in MG. **(A,B)** Confocal microscopy representative images of the chromatin epigenetic landscape in control or NMDA (100 μM/6 h) treated MG, using both repressive (5mC and MeCP2; green) and active (5hmC and H3K4Me3, red) chromatin markers. Calibration bar: 2 μm. Relative fluorescence quantification graphs present data as an unpaired *t*-test, *P < 0.05* **(C,D)** NMDA-treated MG increase Tet3 and multipotency gene expression in culture. Cells were either left untreated or exposed to 100 μM for 6 h. The graphs show the relative expression values of *Tet1, Tet2, Tet3, Gadd45a, Gadd45b* **(C)**, and *Nestin, Ascl1 and Lin28* **(D)** calculated based on the 2^–ΔΔCt^ method, from the Ct data obtained by real-time PCR. Means ± SEM of three different experiments performed in three independent biological replicates are shown (unpaired *t*-test, *P < 0.05).

To evaluate the epigenetic landscape in mouse MG primary cultures, we performed a previously described technique based on the confocal visualization and quantification of the immunofluorescence staining of epigenetic marks associated to transcriptionally inactive methylated DNA (5mC and MeCP2) and transcriptionally active (5hmC and H3K4me3) chromatin (Ramsawhook et al., 2017; Figure 1). DNA and histone methylation marks (5mC, 5hmC and H3K4me3) presented a clear nuclear localization. As previously reported, MeCP2 was immunodetected both in the nucleus and the cytoplasm (Miyake and Nagai, 2007; Figure 1). We applied this technique to evaluate MG epigenetic landscape alterations induced by 6h exposure to 100 μm of NMDA (Figure 2). NMDA induces a 37 ± 10% and 25% ± 4 reduction in the fluorescence intensity of 5mC and MeCP2 signals, respectively (Figures 2A,B). These results may suggest that NMDA induced a transition of the chromatin to a more transcriptionally permissive landscape. This would be further supported by the observation that NMDA induces a 34 ± 12% and 27 ± 7% increase in the fluorescence intensity of 5-hmC and H3K4Me3 labels (Figures 2A,B).

NMDA-induced DNA demethylation could be associated to an increase in the expression of *Tet3*, as evaluated by RT-PCR (Figure 2C) while *Tet1, Tet2, Gadd45a* and *Gadd45b* expression remained unaltered. Furthermore, NMDA exposure induced the expression of the pluripotency genes *Ascl1*, and *Lin2*8, as previously described (Carapia et al., 2023; Figure 2D). These observations suggest that NMDA-induced *Tet3*-mediated DNA demethylation may play a role in the dedifferentiation capacity of Müller cells in response to damage.

### The induction of DNA demethylation is not sufficient to induce pluripotency gene expression in MG

**FIGURE 3:**
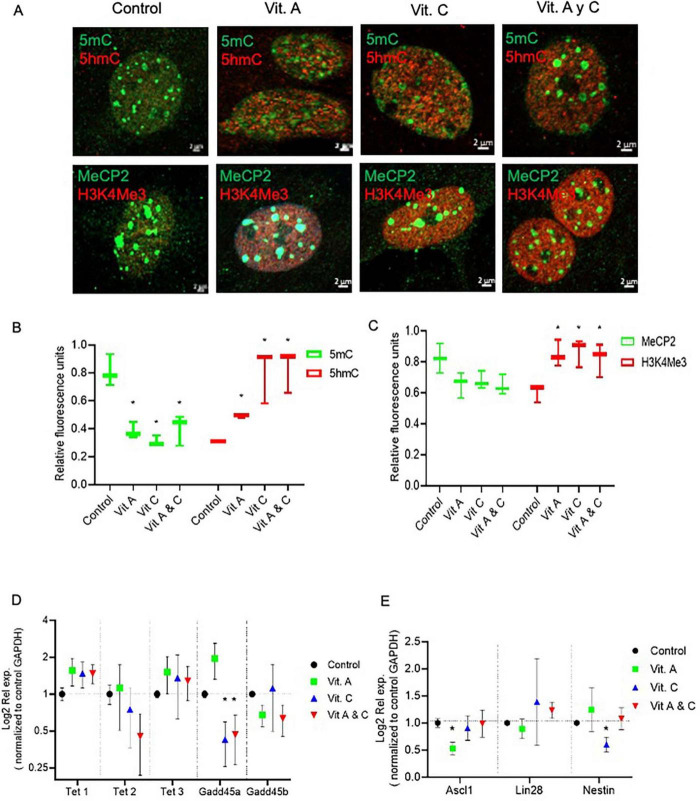
Vitamins A and C did not induce MG dedifferentiation. **(A)** Confocal microscopy representative images of the chromatin epigenetic landscape in control or vitamins A (100 nm) and/or C (100 μM) treated MG, using both repressive (5mC and MeCP2; green) and active (5hmC and H3K4Me3, red) chromatin markers. Calibration bar: 2 μm. **(B,C)** Relative fluorescence quantification graphs present data as Mean ± SEM (One way ANOVA, *P < 0.05). **(D,E)** The graphs show the relative expression values of *Tet1, Tet2, Tet3, Gadd45a, Gadd45b* **(D)**, *and Nestin, Ascl1* and *Lin28* **(E)** calculated based on the 2^–ΔΔCt^ method, from the Ct data obtained by real-time PCR. Means ± SEM of three different experiments performed in three independent biological replicates are shown (One way ANOVA, *P < 0.05).

To assess whether DNA-demethylation was sufficient to induce the dedifferentiation processes in MG, we took advantage of previously reported data demonstrating that vitamins A (Vit. A) and C (Vit. C) regulate Tet enzyme activity (Hore et al., 2016). We treated primary MG cultures with Vit. A, Vit C or a combination of both vitamins and evaluated the epigenetic landscape and the expression of the previously analyzed enzymatic activities and pluripotency genes (Figure 3). At the nuclear level, confocal microscopy visualization and quantification indicated that exposure to vitamins A or C resulted in a 45 ± 15% reduction in 5mC and a corresponding 42% increase in 5hmC (Figures 3A,B). However, none of the treatments altered the fluorescence intensity of the MeCP2 label, and only Vit. C produced a 34% increase in H3K4Me3 (Figure 3C). Furthermore, RT-PCR gene expression analysis demonstrated that none of the Tet enzymes were affected by the vitamin treatments, except for the Gadd45a protein, which showed reduced expression in response to Vit. C (Figure 3D). Next, we examined the effect of vitamins on reprograming genes. None of the genes were induced, *Nestin* expression decreased when Vit. C was the sole stimulus, while *Ascl1* and *Lin28* exhibited reduced expression during Vit. A treatment (Figure 3E).

### Tet3 activity is essential for maintaining the NMDA-induced dedifferentiation capacity of mammalian Müller glia

**FIGURE 4:**
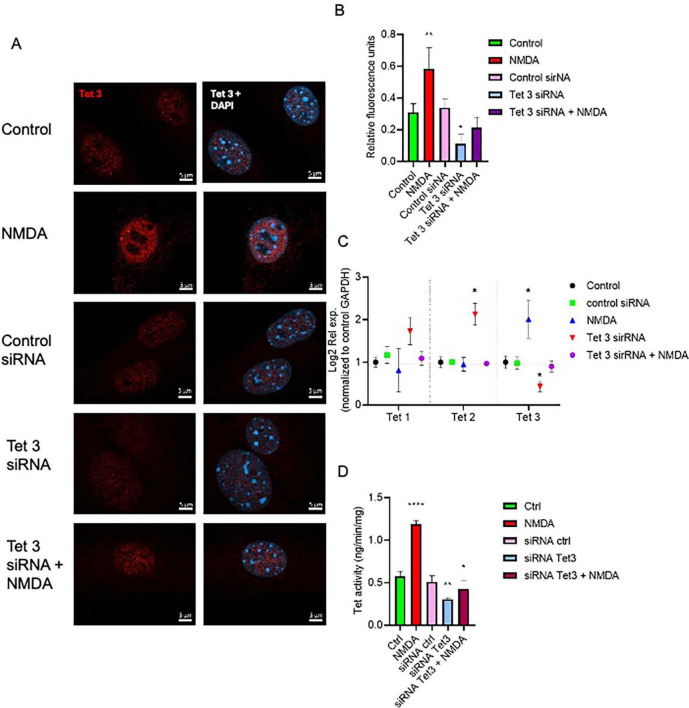
Tet 3 knock down reduces DNA demethylation activity **(A)** Representative confocal microscopy images of Tet 3 nuclear protein expression in MG in response to stimulation with NMDA (100 μM/6 h) and in control or Tet3 siRNA transfected cells 24 h after transfection. **(B)** Relative fluorescence quantification graphs present data as Mean ± SEM (One way ANOVA, *P < 0.05, **P < 0.01). **(C)** Transcriptional effect on Tet enzyme expression calculated based on the 2^–ΔΔCt^ method, from the Ct data obtained by real-time PCR. Means ± SEM of three different experiments performed in three independent biological replicates are shown (One way ANOVA, *P < 0.05). **(D)** DNA demethylation activity assay to measure specific hydroxymethylation as a result of treatments (One way ANOVA, *P < 0.05, ***P < 0.001). Scale bar: 2 μm.

To evaluate directly the role of *Tet3* in the NMDA-induced dedifferentiation capacity of MG we used a specific Tet3 siRNA to block its expression. When analyzing protein expression at the nuclear level (Figures 4A,B), we observed that, consistent with NMDA-induced Tet3 transcription, Tet3 protein levels are also increased following NMDA exposure. Notably, transfection with Tet3 siRNA impairs this NMDA-mediated induction of Tet3 protein. We observed that, while Tet3 siRNA efficiently blocks *Tet3*, it also induces overexpression of *Tet2*, possibly indicating a compensatory effect (Figure 4C). However, when we evaluated pan-Tet DNA hydroxylation activity in MG using a specific enzymatic activity test, we observed that although Tet3 siRNA reduced MG pan-Tet activity by approximately 50 ± 15%, Tet3 deficiency was sufficient to impair the NMDA-induce hydroxylation activity. These observations support a predominant role for Tet3 activity in response to NMDA.

**FIGURE 5:**
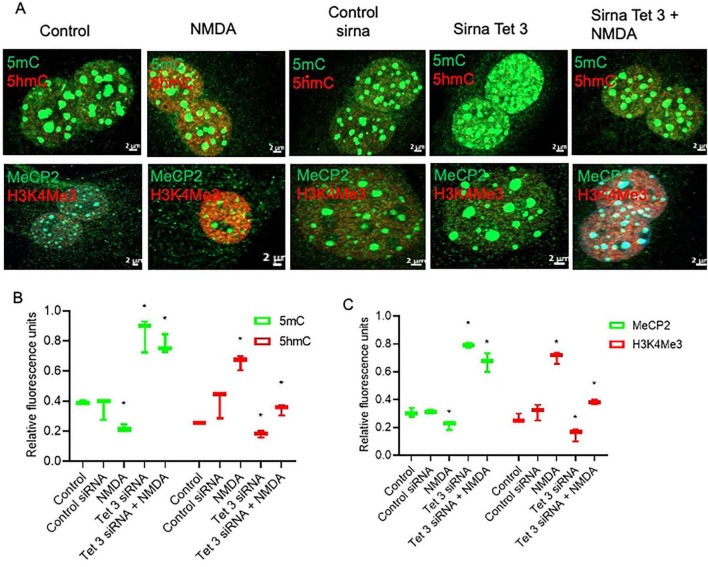
Tet 3 Knockdown induces a repressive chromatin landscape **(A)** Confocal microscopy representative images of the chromatin epigenetic landscape in control, NMDA (100 μM/6 h) and in control or Tet3 siRNA transfected cells 24 h after transfection on MG, using both repressive (5mC and MeCP2; green) and active (5hmC and H3K4Me3, red) chromatin markers. Calibration bar: 2 μm. **(B,C)** Relative fluorescence quantification graphs present data as Mean ± SEM (One way ANOVA, *P < 0.05).

Evaluation of the DNA-methylation associated epigenetic landscapes in Tet3 deficient cells demonstrated altered responses of MG to NMDA (Figures 5A–C). The decreased levels of methylated DNA markers (5mC and MeCP2) that characterized the response to NMDA are reversed in the knockdown cells; we observed a 78% and 64% increase of these markers respectively. Similarly, there is a reversion in the response when we evaluated the unmethylated DNA markers (5hmC and H3K4Me3), instead of an induction driven by NMDA, we observe a 50 ± 11% and 72 ± 9% reduction respectively.

**FIGURE 6:**
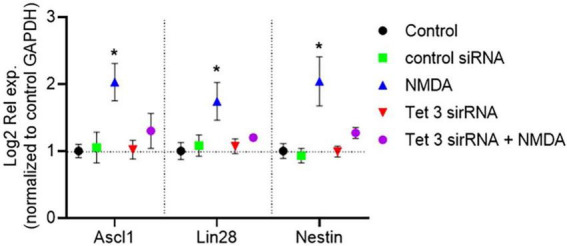
Tet 3 is necessary for inducing the expression of reprograming genes in response to NMDA (100 μM/6 h). The graph shows the relative expression values of *Nestin, Ascl1 and Lin28* calculated based on the 2^–ΔΔCt^ method, from the Ct data obtained by real-time PCR (One way ANOVA, *P < 0.05).

Importantly, RT-QPCR analysis of gene expression demonstrated that siRNA-mediated Tet3 knockdown is enough to prevent the induction of expression of the pluripotency genes *Ascl-1*, *Lin28* and *nestin* in response to NMDA (Figure 6). On the whole, these results revealed an unknown role on Müller glia epigenetic landscape and the dynamics of reprograming genes in the initial response to NMDA that could unlock their further neuronal differentiation capacity.

### Tet 3 overexpression induces an early neural phenotype in MG cultures

**FIGURE 7:**
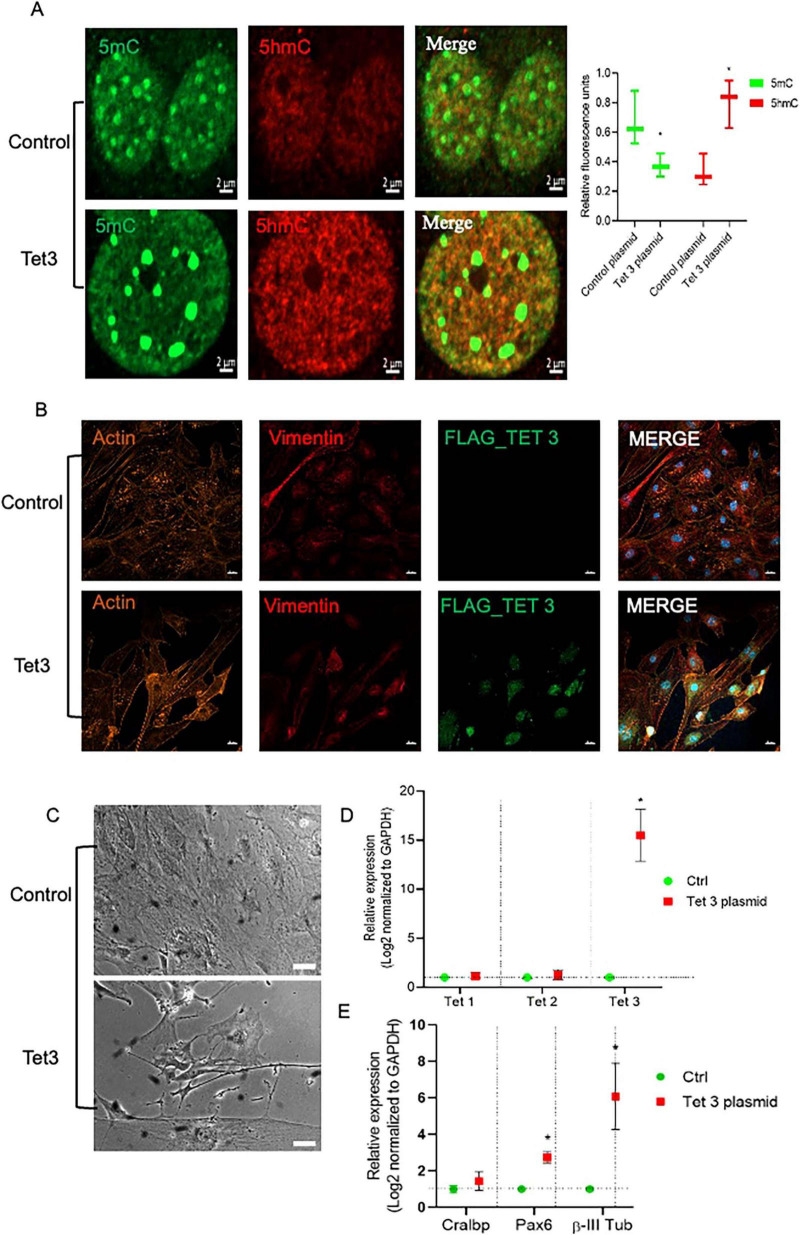
Tet 3 overexpression induced DNA demethylation, morphological and transcriptional changes in cultured MG. **(A)** Representative images of confocal imaging of immunofluorescence to assess the chromatin epigenetic landscape in cultured MG, using both repressive (5mC, green) and active (5hmC, red) chromatin markers. Calibration bar: 10 μm. Relative fluorescence quantification graphs present data as an unpaired *t*-test, *P < 0.05*. **(B)** Representative confocal microscopy images of cultured MG 7 days after transfection of a control or a FLAG-tagged Tet3 overexpression plasmid using actin and vimentin immunolabeling for morphological references, and FLAG immunolabeling as a Tet 3 plasmid transfection indicator. **(C)** Bright field imaging showing morphological differences between control and Tet 3 transfected cells. **(D)** Tet 3 overexpression confirmed by qPCR; calculated based on the 2^–ΔΔCt^ method, from the Ct data obtained by real-time PCR. **(E)** Assessment of lineage markers; Cralbp, Pax6, βIII tubulin by qPCR; calculated based on the 2^–ΔΔCt^ method, from the Ct data obtained by real-time PCR. Calibration bar: 20 μm. (One way ANOVA, *P < 0.05).

**FIGURE 8:**
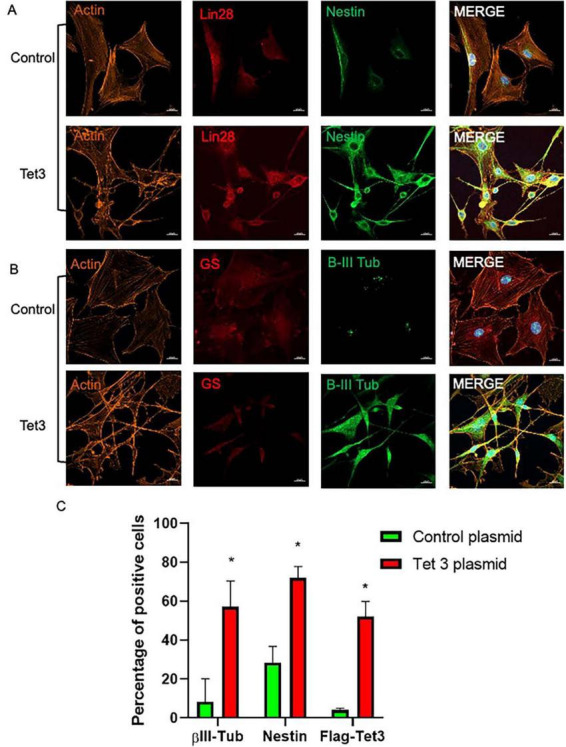
Characterization of Tet 3 overexpressing MG. **(A)** Representative confocal microscopy images of cultured MG 7 days after transfection of a control or a Tet3 overexpression plasmid using an actin antibody for morphological reference, and Lin28 and Nestin antibodies to assess progenitor gene expression. **(B)** Representative confocal microscopy images of cultured MG 7 days after transfection of a control or a Tet3 overexpression plasmid using actin as morphological reference and Glutamine synthase (Gs) and βIII-tubulin to assess glial or neural nature of cells. **(C)** Percentage of immunopositive cells. Scale bar: 20 μm. (One way ANOVA, *P < 0.05).

To better understand the scope of Tet3 enzymatic activity in the dedifferentiation and neurogenic capacity of MG we used an overexpression system based on a Tet3 flag-tagged plasmid. Tet3 overexpression induced 63% decrease in 5mC while inducing a significant increase in 5hmC (Figure 7A). The transfected cells were tested by immunofluorescence to confirm the presence of the flag-tag 48 h after transfection while, as expected, the expression of Actin and Vimentin was not significantly altered by the procedure (Figure 7B). We observed that cells transfected with the control plasmid exhibited the typical Müller glia morphology in a confluent state, while those transfected with the Tet3 plasmid displayed a morphology resembling neural progenitor cells, characterized by a more elongated shape and numerous projections (Figure 7C). The transcriptional profiles confirmed Tet3 overexpression (Figure 7D) without affecting other Tet isoforms. We then assessed the expression of Cralbp as a glial marker, b-III Tubulin as an early neural marker, and Pax6 as a neural marker (Figure 7E). Cralbp expression remained unchanged between treatments, while Pax6 levels increased approximately twofold compared to the control plasmid. Notably, βIII-tubulin showed a sixfold increase relative to control. In addition to morphological changes, immunofluorescence analysis of transfected cells showed that Tet3 overexpression increased Nestin and b-III Tub immunolabeling while Lin28 remained unchanged, and GS decreased a 36 ± 9% (Figure 8).

## Discussion

In this study, we demonstrate that an initial wave of DNA demethylation is essential for preserving the dedifferentiation capacity of mammalian Müller glia in response to high concentrations (100 μM) of NMDA. While retinal neuronal NMDA receptors are known to mediate glutamate-induced neurodegeneration (Boccuni and Fairless, 2022), NMDA can also trigger MG reprograming, leading to the generation of new bipolar, photoreceptor, or amacrine cells (Bringmann et al., 2004; Karl et al., 2008). Building on previous findings that NMDA induces the expression of pluripotency genes such as *Nestin*, *Ascl1*, and *Lin28* in MG cultures (Takeda et al., 2008; Reyes-Aguirre et al., 2013; Xiao et al., 2017; Carapia et al., 2023), we show that NMDA specifically upregulates Tet3 at both the mRNA and protein levels. This is accompanied by a shift in the chromatin landscape from a transcriptionally restrictive, DNA-methylated state to a more permissive, DNA-unmethylated configuration. Moreover, our findings reveal that Tet3-driven DNA demethylation is crucial for sustaining the dedifferentiation potential of mammalian Müller glia.

DNA demethylation has been extensively associated with the reactivation of pluripotency genes, which are hypermethylated and silenced in somatic cells, during the reprograming processes of multiple cell types (Lee et al., 2014; Ghazimoradi and Farivar, 2020). In the retina, where the Tet-dependent regulation of 5hmC formation is essential for developmental neurogenesis (Seritrakul and Gross, 2017; Heilman et al., 2025), Tet3 has been shown to accumulate in retinal neurons over time (Perera et al., 2015). Retinal regeneration after damage does not necessarily recapitulate events that characterize retinal development. However, it has been demonstrated that genes involved in the DNA methylation/demethylation processes exhibit altered expression patterns in dedifferentiating MG cells in both zebrafish and mice (Powell et al., 2013; Reyes-Aguirre and Lamas, 2016). Furthermore, it has been also demonstrated that Tet3 is sufficient to reprogram retinal pigment epithelium (RPE) cells in the regenerating chicken retina (Luz-Madrigal et al., 2020). In the present study, we demonstrate, for the first time, that, specifically, Tet3 deficiency impairs the dedifferentiation capacity of rodent Müller glia.

We observed that Tet3 knockdown induced both the transcriptional and protein expression of Tet2, suggesting a compensatory effect similar to that reported in Tet1 knockout mice (Kumar et al., 2015). However, the lack of induction of pluripotency gene expression, even under conditions of elevated Tet2 expression, supports a distinct, non-overlapping role for Tet3 in the acquisition of a progenitor-like phenotype in Müller cell cultures. Furthermore, we demonstrate that an initial wave of demethylation is essential but not enough to induce the dedifferentiation capacity of these cells. Thus, the acquisition of a DNA demethylated landscape through exposure to vitamins A and C was neither associated with an increase in Tet3 expression nor to a transcriptional change in the genes associated to MG dedifferentiation. Interestingly, it has been reported that vitamins A and C, in combination, could reprogram embryonic stem cells via a DNA demethylation mechanism (Hore et al., 2016). Our results point out that DNA demethylation must be directed to specific transcriptional targets to achieve cell reprograming. In contrast to vitamin exposure, NMDA treatment in MG resulted in increased levels of 5hmC and its downstream marker H3K4me3, along with a reduction in 5mC and MeCP2 levels. The specific DNA sites or genes affected by these modifications, including possible sites in the *Lin28*, *Ascl1* and *Nestin* promoters, are out of the scope of this work. However, it has been reported that MeCP2 suppresses the expression of Lin28 via transcriptional control (Xu et al., 2016; Kim et al., 2019). We could speculate that, in NMDA-exposed MG, a DNA unmethylated landscape prevents the binding of MeCP2 to *Lin28* and favors the dedifferentiation response. Tet3, on the other hand, has been previously associated with NMDA signaling in various cellular models. Direct NMDA stimulation of neurons has been shown to rapidly induce Tet3 transcription (Kremer et al., 2018). Furthermore, Tet3 is essential for memory, cognition, and fear responses, all of which are linked to NMDA signaling (Li et al., 2014; Yu et al., 2015).

To date, few studies have explored the potential interactions between Tet3 and key neural stem cell markers such as *Ascl1*, *Lin28*, and *Nestin*. It has been demonstrated that Tet 3 works in a self-regulatory axis with Let7 and Lin28 to maintain stemness in pancreatic cancer cells (Liu et al., 2020). Additionally, Tet3 is regulated by a member of the miRNA-200 family, which controls the proliferation and differentiation of olfactory globose cells (Yang et al., 2020). The miR-200 family has also been implicated in modulating the regenerative response of Müller glia, suggesting that these miRNAs could potentially act as transcriptional regulators of Tet3 (Jiang et al., 2024).

Interestingly, it had been previously reported that Tet 3, along with other facilitating cofactors, could convert mouse embryonic fibroblasts directly to neurons through a DNA demethylation pathway (Zhang et al., 2016) and extensive DNA demethylation has been observed in primary neurons (Yu et al., 2015). We speculated that over-expression of Tet 3 on MG cells could ease the glia to neuron or neural progenitor cell transition. Our results demonstrated that Tet3 contributed to morphological changes and to the induction of expression of neuronal progenitor (Nestin) and early neuron (β-III Tub) genes in more than 50% of the cells. Additionally, when analyzing MG marker genes, we observed that while the level of expression of GS decreased in Tet3 overexpressing cells, the level of expression of CRALBP remained constant between control and Tet3 cells. These observations suggest that the reprograming process was not completed. The complete transition to a postmitotic neuronal state, if achievable, may require prolonged Tet3 expression or the application of metabolic inducers that facilitate reprograming. Furthermore, unlike the transient transfection system we used, a stable knockout system could provide more accurate insights into the role of Tet3 in NMDA-treated cells, overcoming the limitation of partial effects due to uneven transfection. Overall, our findings build upon existing evidence supporting the crucial role of epigenetic mechanisms in the limited regenerative capacity of mammalian Müller glia and may offer new therapeutic targets, such as Tet3, for a range of retinopathies.

## Data Availability

The raw data supporting the conclusions of this article will be made available by the authors, without undue reservation.

## Footnotes

1
http://n2t.net/addgene:60940
